# Orally Administered Fumonisins Affect Porcine Red Cell Membrane Sodium Pump Activity and Lipid Profile without Apparent Oxidative Damage

**DOI:** 10.3390/toxins12050318

**Published:** 2020-05-12

**Authors:** András Szabó, Omeralfaroug Ali, Katalin Lóki, Krisztián Balogh, Miklós Mézes, Tibor Bartók, Levente Horváth, Melinda Kovács

**Affiliations:** 1 “MTA-KE-SzIE Mycotoxins in the Food Chain” Research Group, Hungarian Academy of Sciences-Kaposvár University, 7400 Kaposvár, Hungary; kovacs.melinda@ke.hu; 2 Faculty of Agricultural and Environmental Sciences, Kaposvár University, 7400 Kaposvár, Hungary; omeralfaroug.ali@gmail.com (O.A.); loki.katalin@ke.hu (K.L.); 3 Department of Nutrition, Faculty of Agricultural and Environmental Sciences, Szent István University, 2013 Gödöllő, Hungary; balogh.krisztian@mkk.szie.hu (K.B.); mezes.miklos@mkk.szie.hu (M.M.); 4 Fumizol Ltd., 6725 Szeged, Hungary; tibor.bartok@fumizol.hu (T.B.); levente.horvath89@gmail.com (L.H.)

**Keywords:** fumonisins, red blood cell, sodium pump, membrane, fatty acids, oxidative stress, pig

## Abstract

Weaned piglets (*n* = 3 × 6) were fed 0, 15 and 30 mg/kg diet fumonisin (FB_1_, FB_2_ and FB_3_, i.e., FBs, a sphinganine analogue mycotoxin), from the age of 35 days for 21 days, to assess mycotoxin induced, dose-dependent changes in the red cells’ membrane. Ouabain sensitive Na^+^/K^+^ ATPase activity was determined from lysed red cell membranes, membrane fatty acid (FA) profile was analysed, as well as antioxidant and lipid peroxidation endpoints. Final body weight was higher in the 30 mg/kg group (vs. control), even besides identical cumulative feed intake. After 3 weeks, there was a difference between control and the 30 mg/kg group in red cell membrane sodium pump activity; this change was dose-dependent (sig.: 0.036; R^2^ = 0.58). Membrane FA profile was strongly saturated with non-systematic inter-group differences; pooled data provided negative correlation with sodium pump activity (all individual membrane n6 FAs). Intracellular antioxidants (reduced glutathione and glutathione peroxidase) and lipid peroxidation indicators (conj. dienes, trienes and malondialdehyde) were non-responsive. We suppose a ceramide synthesis inhibitor (FB_1_) effect exerted onto the cell membrane, proven to be toxin dose-dependent and increasing sodium pump activity, with only indirect FA compositional correlations and lack of lipid peroxidation.

## 1. Introduction

Fumonisins are mycotoxins (fungal secondary metabolites) produced in the highest quantities by *Fusarium verticillioides* and *Fusarium proliferatum* mould strains, infecting food and feed cereals. The 28 fumonisin analogues characterized since 1988 can be divided into four main groups: series A, B, C and P [1], from which the B analogues are toxicologically the most hazardous, fumonisin B_1_ (FB_1_) being the most well-known and the most toxic in the latter series [2]. Fumonisin occurrence is very frequent in cereals and cereal products but primarily in corn; the prevalence was 78% in 2020 in the tested corn samples [3], representing the main farm animal feed component. Fumonisins are specifically harmful to pigs, leading to a typical porcine toxicosis syndrome named porcine pulmonary edema. Hepatic lesions consisting of apoptosis, necrosis and hepatocyte proliferation, besides elevated serum cholesterol concentration are the further consequences. In chronic studies, oesophageal plaques, hyperplastic hepatic nodules and right ventricular hypertrophy were found in pigs as well [4].

At a molecular level, fumonisin B_1_ administration disrupts sphingolipid biosynthesis, with the greatest alterations in sphingosine and sphinganine concentrations in porcine kidney, liver, lung and heart [4,5]. FB_1_ shows structural similarity to the cellular sphingolipids and inhibits ceramide synthase, leading to the accumulation of sphinganine and depletion of ceramide [6]. Fumonisin B_1_ is so potent and so specific in this regard that it is referred to as a direct ceramide synthesis inhibitor [7]. In in vitro exposures, there is a quick increase in the free sphingoid base, sphinganine [8], while sphinganine acylation (with fatty acids) is also inhibited.

The ceramide synthesis inhibitor effect has thus been proven in vitro [8] and in vivo in numerous animal species and multiple tissue types [4]. According to a recent review [2], FB_1_ has been shown to produce pleiotropic toxicities in animals, including neurotoxicity, hepatotoxicity and nephrotoxicity, and recently we proposed haematotoxicity [9]. Underlying mechanisms include disrupted sphingolipid metabolism, oxidative stress, activation of endoplasmic reticulum stress, modulation of autophagy and the alteration of DNA methylation [2].

These systematic effects have also consequences on the ion balance and its regulation. Haschek et al. (1992) [10] described in short-term cardiovascular studies decreased cardiac contractility, mean systemic arterial pressure, heart rate and cardiac output due to FB_1_ and increased mean pulmonary arterial pressure, the changes being compatible with the inhibition of L-type calcium channels by increased sphingosine and/or sphinganine concentration. In 2014, we [9] described dramatically increased cation flux (Na^+^, K^+^) in FB_1_ fed rabbits’ erythrocytes, the result being fully consonant with those of Mays et al. (1995) [11], reporting that Na^+^/K^+^ ATPase sorting to the different membrane domains (in renal cells apically and baso-laterally) is modified by the inhibition of the sphingolipid synthesis, the typical FB_1_ mode of action. Thus, FB_1_ exerted its sodium pump modification effects definitely by altering the lipid synthesis, and not directly interfering with Na^+^/K^+^ ATPase enzyme itself.

Based on the recent results, and the continuously increasing fumonisin burden, we aimed to study whether weaned piglets are susceptible to a graded dietary FBs exposure (dose dependence), testing circulating red cell membrane FA composition, Na^+^/K^+^ ATPase activity and oxidative stress indicators, after in vivo exposure.

## 2. Results

### 2.1. Body Weight, Organ Masses

#### 2.1.1. Inter-Group Differences and Dose Response

Final body weight (BW) was significantly higher in the 30 mg FBs/kg group, as compared to the control, but absolute organ mass mean values were not different among any of the groups, as compared with analysis of variance (Table 1). The relative kidney weight was the lowest in the 30 mg/kg group and significantly different from the control. Cumulative feed intake for the entire 21 days was identical in the three groups (Table 1). The only somatic trait that was found to be linearly related to mycotoxin dose was the final BW (sig.: 0.027; R^2^ = 0.27).

#### 2.1.2. Sodium Pump Activity Correlations

The somatic traits determined did not provide any significant correlation (Pearson correlation) with the RBC sodium pump activity.

### 2.2. Red Cell Membrane Sodium Pump Activity and Dose Response

#### Inter-Group Differences and Dose Response

**Figure 1:**
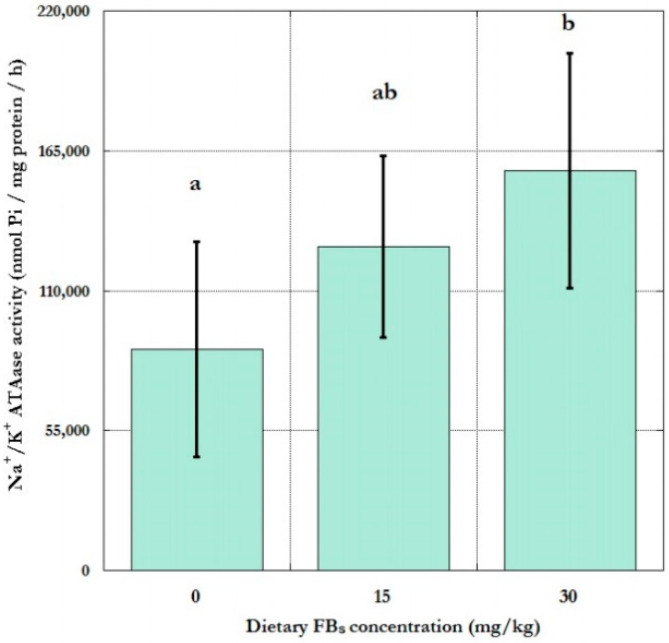
The total Na^+^/K^+^ ATPase activity of red cells of the experimental piglet groups (*n* = 6/group; columns represent group means ± SD of 6 individual data; different uppercase letters indicate significant difference of means at *p* ≤ 0.05. Between group differences were compared with one-way ANOVA and LSD “post hoc” test).

After 5 weeks of FBs feeding, there was a difference among the control and the 30 mg/kg groups in the red cell membrane sodium pump activity (Figure 1). The FBs feeding increased the sodium pump activity significantly only in the latter group, while the 15 mg/kg treatment provided intermediate data (control < 15 < 30), the latter without statistical significance. Testing the dose response of this alteration, the linear estimation was significant (y intercept = 52,982; slope = 35,340; sig.: 0.036; R^2^ = 0.58).

### 2.3. Red Cell Fatty Acid Profile

The membrane fatty acid profile is summarized in Table 2, also showing inter-group differences and the dose-response data.

#### 2.3.1. Inter-Group Differences and Dose Response

The proportion of C12:0 (lauric acid) was the highest in the control group and the lowest in the 15 mg/kg treatment, the 30 mg/kg showing intermediate values.

Clear toxin dose dependent difference in the fatty acid proportions was found for the following FAs: C18:1 n9 (oleic acid), C20:3 n6 (dihomo-γ-linolenic acid), C20:4 n6 (arachidonic acid) and total monounsaturation (MUFA), 15 mg/kg group showing higher data than the 30 mg/kg treatment. In all these instances the control group had intermediate proportional values. The only compound for which the 15 mg/kg group had the lowest proportion was C16:0 (palmitic acid), and as a direct consequence, the sum of saturated FAs. As a consequence of this non-linear alteration mode, well-fitting linear dose-response was not proven in any of the cases.

#### 2.3.2. Sodium Pump Activity Correlations

The Pearson correlation between the membrane fatty acid proportions and the sodium pump activity values is given in Table 3, for the description of the inter-relationship between the variables. Practically all individual n6 FAs provided negative correlation with the sodium pump activity, as well as the total polyunsaturated FAs (PUFA).

### 2.4. Red Cell Antioxidant Status and Lipid Peroxidation

**Figure 2:**
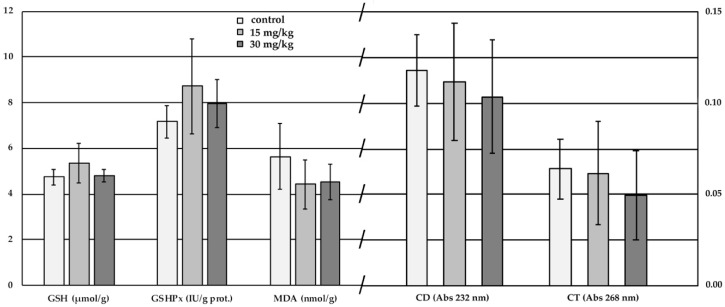
The antioxidant and lipid peroxidation traits of the red cells of the experimental piglet groups (lack of different uppercase index letters represent the lack of significant difference at *p* < 0.05). (*n* = 6/group; columns represent group means ± SD of 6 individual data. Between group differences were compared with one-way ANOVA and LSD “post hoc” test).

Whole red cell homogenate reduced glutathione (GSH), glutathione peroxidase (GSHPx), conjugated diene and triene concentrations (CD and CT), and malondieldyde (MDA) concentration did not provide any inter-group differences (Figure 2), neither linear dose-response, nor any correlations with the sodium pump activity.

## 3. Discussion

### 3.1. Body Weight, Organ Masses

#### Inter-Group Differences and Dose Response

The FBs administration level was relatively high in this study, being definitely above the limit values for pigs [12]. The study planning aimed to reach or approach an intoxication status/niveau that is not mild, primarily to affect the cellular composition of the already circulating, ripe red cells [9,13] and to test possible mediator effect of plausible oxidative stress. Though the initial BW was equal in all groups, the highest fumonisin dose increased it, as compared to the control. This increase was not matched with the increase of any of the organ mass values (and was neither a result of increased feed intake, Table 1). In contrast, final BW provided linear fumonisin dose dependence (sig.: 0.027; R^2^ = 0.27, as tested on individual data-pairs). (Moreover, by testing weekly BW gain in the three groups separately, it was clearly visible that gain slowed down, and its standard deviation (as assessed with Levene’s F test) increased markedly, in parallel (data not shown)).

Generally, FB_1_ is responsible for the induction of either organ, or even body mass alterations. We have reported [14] that the liver mass decreased, kidney mass increased and BW increased due to 5 and 10 days of FB_1_ exposure at 0, 20, 50 and 100 mg/kg dietary dose equivalent, though in other cases no alteration was found in rats [15]. Furthermore, in rabbits, liver mass was found to increase as a result of 10 mg FB_1_/kg diet exposure for 4 weeks [16]. Specifically, for pigs, FB_1_ has been reported as a growth inhibitor [17], most probably by damaging the barrier function of the intestinal epithelial cells [18]. In contrast, in an approximation of the limit values [12], histological symptoms in intestine and myocardium appeared at 3.7 mg FBs/kg and in the kidney at a higher dose (8.1 mg/kg feed), while at the highest feed dose (12.2 mg FBs/kg) all investigated organs showed histological modifications, primarily lesions. In the above study [12], 12.2 mg/kg diet did not lead to growth differences, nor the 20 mg FB_1_/kg for 10 days could alter the performance, body weight and feed intake in another test [13]. Since our present study strongly exceeded the cited mycotoxin concentrations [12] and exposure period [13], we assumed that increased growth of the mostly intoxicated animals was attributable to some extent of slight edema [13], although absolute lung weight was not altered statistically. It is important to add that pulmonary edema was present in some individual cases, was photo-documented, but this was not a systematic finding. In this study, we did not confirm any alteration of the absolute organ weights, while total body mass increased. Ultimately, the daily and cumulative feed intake (Table 1) and feed conversion ratio were as well checked, not providing any inter-group differences (data not shown). Therefore, we assume that the applied dose did not compromise the piglet performance, at least under the examined period, whereas the recorded increase in body mass of the 30 mg/kg diet group is a slight increase that might be a result of non-systematic edema that not detected in all animals. Results of the biochemical analysis (higher enzymatic activities of LDH, γ-GT and CK for 30 mg/kg diet, data not shown) are indirectly indicating possible slight muscular hypertrophy, in which increase in body mass is the major consequence. This later assumption cannot be proven in this study since the muscular mass was not examined.

### 3.2. Red Cell Membrane Sodium Pump Activity and Dose Response

Similarly to our first report [9] on the disturbance of cation active transport as induced by FB_1_, we found a strongly similar result now, but in a species never tested for this reaction before. Which fumonisin modified pathways/compounds are exactly involved in the dose-dependent of the RBC active cation transport has not been elucidated yet.

Early in vitro studies revealed that FBs are effective inhibitors of sphinganine (sphingosine) N-acyl transferase (ceramid synthases, CerS) [19]. By in vitro exposure, when cells are exposed to FB_1_, there is a quick increase in the free sphingoid base, sphinganine, in tissues and body fluids [20], while sphinganine acylation is inhibited. Moreover, it has been added [21] that FB_1_ inhibits as well the production of 1-deoxydihydroceramide, playing important roles in cellular level regulation.

The well-documented biological effect of FB_1_ is thus a more complex inhibition of sphingolipid synthesis, leading to cellular ceramide level depletion (blocking the acylation by FAs). Evidence exists that ceramide effectively modulates Na^+^/K^+^ ATPase enzyme activity (and that of further sodium transporters), participating in its regulatory network in the renal cells’ basolateral membrane [22]. Moreover, increased ceramide levels induce ouabain sensitive sodium pump inhibition [22] but indirectly; this happens in renal cells via the modulation of protein kinases A and C. Ceramide can have a crucial role in the regulation of the complex Na^+^ transport apparatus (directly inhibiting furosemide sensitive Na^+^-ATPase but indirectly inhibiting ouabain sensitive Na^+^/K^+^ ATPase), involving also effector proteins [22]. This supposed modulatory role (possessing most (~60%) of the Na^+^/K^+^ ATPase inhibitory activity) of ceramide onward Na^+^/K^+^ ATPase is a novel finding in the red cells. In the present dataset, gained from a sustained FB_1_ intoxication study, (FB_1_ is known as an inhibitor of ceramide synthesis) strongly elevated RBC sodium pump activity was proven after 3 weeks of exposure.

Ceramide has the property to act as a regulator of multiple cellular processes, like cell proliferation and apoptosis, which are coupled respectively with decreasing and increasing the Na^+^/K^+^ ATPase activity (in nucleated cells) [23]. These antagonistic effects might be time-dependent, due to different signalling pathways and their different activation time intervals. Since apoptosis is a process characterized by cell volume shrinkage, and it has a direct influence on cellular ion exchange dynamics, those (more specifically K^+^) are indeed triggering factors of apoptotic processes [24], as found in lymphocytes. This K^+^ homeostasis modification is merely a result of Na^+^/K^+^ ATPase activity change [23]. Anyway, ouabain sensitive sodium pump is ubiquitous and has multiple triggering factors, like hormonal changes, substrate concentration, as well as the embedding membranes’ physicochemical properties [25,26].

Specifically for human erythrocytes, the enzyme stimulatory effect of ceramide (for Ca^2+^-ATPase) was presented [27]. An interesting property of this Ca^2+^-ATPase that distinguishes it from other P-type ionic pumps is the multiplicity of its regulatory mechanisms [28]. Authors found that ceramide acts on the enzyme activity in its second messenger role and not by influencing membrane or caveola properties. HepG2 cells react to in vitro ceramide addition with increased Na^+^/K^+^ ATPase activity [23]. It seems that ceramide provides an enzyme activating effect on P-type ionic pumps, but the ceramide metabolite, sphingosine is in lack of this property; this effect has been reported to be time-dependent [23].

In summary, we found a speed-up of the most important cations’ influx (Na^+^) and efflux (K^+^) in the porcine red cells, as induced by a ceramide-synthase inhibitor, and newly, providing inhibitor dose-dependence.

### 3.3. Red Cell Fatty Acid Profile

#### 3.3.1. Inter-Group Differences, Dose Response and Sodium Pump Activity Correlations

The Na^+^/K^+^ ATPase inhibition/lowered activity is mostly characteristic for apoptosis, while activity increase is generally a sign of cell proliferation, as reported in hepatic cells [29,30,31]. We analysed mixed, circulating red cell populations and did not check haematology (neither polychromatic erythroblasts). While in our first study [9] FB_1_ exerted clearly defined effects on the rabbit RBC membrane FA composition, here we mostly detected differences between the lower and the higher toxin doses. If control data are considered as a baseline, lower and higher FBs doses exerted likewise divergent effects on the FA profile. It is hard to explain this, since it looks like lower FB_1_ dose has opposite effects than the higher. If focusing specifically on the divergent alterations provoked by the two doses (lower vs. higher), we detected a definitely lower niveau of membrane saturation (lower level of C16:0, higher C18:1 n9 and C20:4 n6, lower total saturation and higher total monounsaturation). These modifications are unequivocally referring to a more rigid physicochemical property of the cell membrane, associated with the higher FBs dose.

In summary, it rather seems that the biological compound, the cellular membrane itself, was an originally less sensitive system in terms of lipid composition towards lipid peroxidative damage. We assume that further studies are needed to clarify the contribution of FAs to the supposed regulatory role of Na^+^/K^+^ ATPase. Most probably, these studies need to separate lipid classes before FA analysis into more relevant sub-classes.

#### 3.3.2. Sodium Pump Activity Correlations

The Pearson correlation between the membrane fatty acids and the sodium pump activity is given in Table 3. Nearly all individual n6 FAs proportional value provided negative correlation with the sodium pump activity, as well as the total polyunsaturated fatty acids (PUFA).

Since Na^+^/K^+^-ATPase is an intrinsic membrane protein, the physico-chemical properties of the membrane constituents should be an important determinant of enzyme activity [32]. Free fatty acids, or those released from the membrane by phospholipase A_2_ (PLA_2_) tend to inhibit the Na^+^/K^+^ ATPase [32], but we did not detect drastic membrane PL disruption for the fatty acid containing lipids. Indeed, this was underscored by the membrane FA compositional results as well, since only mild changes were proven.

Though we measured whole cell membrane, data of Else et al. (2003) [33] in a different membrane model partly supported our data. Though we did not find any positive correlation with the long-chain, n-3 FAs [33], we provided evidence that nearly all n-6 FAs acted as sodium pump activity “slowing down agents” (Table 3). This is consonant with the cited results [33], but it is rather novel that in our, basically small dataset all n-6 series FA were negatively correlated with the sodium pump activity.

There is still a debate on the role of FAs in the regulation of sodium pump. Considering non-ester bound, long chain acids (C16:0, C18:1 n9 and C18:2 n6), those were reported to be very efficiently incorporated to the human erythrocyte membrane, but there, they still remained free fatty acids and did not alter the molecular activity of the enzyme [34]. In a next step, the same authors found [35] lower incorporation efficacy of phosphatidylcholine-FAs (PC-FA) but reported PC-FA dependent decrease (C12:0, C14:0 and C16:0) of the sodium pump. Results prove that the sodium pump in situ is sensitive to lipid fatty acid profile. Our dataset proved this as well, but it has to be added that our analytical approach was only assessing FA methyl esters liberated from an ester bond, thus excluding all free FAs [36].

In summary, we suppose that only a minor toxic effect was attainable in the circulating porcine red cell cohort; fatty acid proportions and enzyme activity data were only loosely related, meanwhile the entire dataset was proving some basic inter-relationship between red cell membrane composition and FA profile. Though only indirectly conceptualized, the basic effect of FBs onward the ceramide synthesis and its consequences seemed to be effectively proven in our data as well, in a species not tested yet in this aspect.

### 3.4. Red Cell Antioxidant Status and Lipid Peroxidation

Analysing early phase (conjugated diene and triene) and progressive (MDA) lipid peroxidation, as well as cellular antioxidant (reduced GSH) concentration and antioxidant enzyme (GSHPx) activity underscored the full lack of lipid peroxidation and antioxidant depletion in this study, in this tissue type. In our earlier relevant study [9], we did not check lipid peroxidation in the homogenized red cells.

Direct incorporation of FB_1_ sensitizes PC bilayer to lipid peroxidation [37], but a metabolized form of FB_1_ is not always found to be oxidative. In some of our earlier studies, we frequently tested FB_1_ induced oxidative stress in red cell homogenate, but only in a very early approach, at 45 mg FB_1_/kg feed dose for 10 days, piglets provided lower reduced glutathione content in the red blood cell haemolysate samples [38].

The erythrocyte is a unique biological structure containing generally high PUFA amounts, molecular oxygen and ferrous ions in the ligand state. For these reasons, it might be expected to be highly vulnerable to the main potential hazard of an aerobic environment [39]. Indeed, due to these cellular properties, the lack of nucleus and especially mitochondria, dramatic lipid peroxidation was not expected. The primary site of possible lipid peroxidation is thus the membrane. The general lack of peroxidative damage that was found here might be attributed to the overall high level of saturation of the porcine red cell membrane (Table 2).

In summary, the ghost cell membrane preparation seemed to result in a relatively high FA saturation, most probably preventing lipid peroxidation. This was in full agreement with the lack of lipid peroxidation and lack of alteration in the antioxidant defence system, as analysed in intact, whole red cells.

## 4. Conclusions

We postulate that red blood cell damage or functional modification is efficiently achieved by fumonisin Bs in piglets after only three weeks. The significantly elevated sodium pump activity is with the highest plausibility induced by the ceramide synthesis inhibitor effect of FB_1_. This mycotoxin leads to a characteristically non-oxidative stress associated state with markedly elevated sodium pump activity and complex membrane lipid fatty acid profile, providing relevant correlations for all n-6 fatty acids.

## 5. Materials and Methods

### 5.1. Experimental Design and Animals

Altogether 3 x 6 weaned Danbred piglets were enrolled in the study at the age of 35 days. After a 14-day adaptation (at the exact age of 49 days) period, the duration of the feeding trial was 21 days. One group was fed a piglet diet complemented with 15 mg/kg FB toxins (FB_1_, FB_2_ and FB_3_ in a fungal culture), whereas another 30 mg/kg (the control was FBs free). Detailed diet composition is given in Table 4. Water was offered ad libitum. The piglets were caged individually in 80 x 80 cm area metabolic cages. The temperature of the trial room was controlled in accordance with the needs of weaned piglets. Bodyweight was measured individually. At the end of the trial, the piglets were euthanised by exsanguination after sedation (Euthanyl-Pentobarbital Sodium, 400 mg/mL, Dechra Veterinary Products, Shrewsbury, UK), and splanchnic organs and blood were sampled.

### 5.2. Feed Mycotoxin Contamination

The basic feed was of commercial origin. Feed was given twice a day, in two equal portions, and the amount of feed not consumed by the animals was measured back. A *Fusarium verticillioides* fungal culture of high FB_1_ concentration (for production details see: [40], culture name: RL 596) was mixed into the ration of the experimental animals, so as to provide a daily FBs (FB_1_+FB_2_+FB_3_) feed concentration of 15 and 30 mg/kg. The mycotoxin concentration of the control and the experimental feed was determined with LC-MS (Shimadzu, Kyoto, Japan). The limit of detection (LOD) for FB_1_ was 3 µg/kg. The diet fed to the control group did not contain detectable amounts of FBs (the full absence of deoxinivalenol, zearalenone and T-2 toxin was as well controlled and confirmed).

Fumonisin B contents were determined with a LC-MS method, described in detail earlier [41].

### 5.3. Blood Sampling, Erythrocythe “Ghost” Preparation

Fresh venous blood was sampled into heparinized (20 IU/mL whole blood) tubes and was centrifuged for 10 min at 1000 g (SIGMA 3-30KS refrigerated centrifuge, Osterode am Harz, Germany). Plasma and the buffy coat were removed, and the erythrocyte bulk was washed 3 times with 10 volumes of TRIS-HCl (0.1 M; pH = 7.4) at 4 °C. After each wash, the buffy coat (and the washing medium as supernatant) was siphoned. Red blood cell (RBC) lysis was induced by ice-cold hypotonic TRIS-HCl solution (15 mM; pH = 7.4). Erythrocyte lysate was centrifuged at 15,000 g for 10 min at 4 °C repeatedly, until the washing medium was colorless (~7 times). Washing medium hemoglobin content was controlled with spectrophotometry at 418 nm in 10 mm path length optical cuvettes against medium blank until 0.001 Abs value (Shimadzu UV160 spectrophotometer, Shimadzu, Kyoto, Japan). The original method was described by Shanmugasundaram et al. (1992) [42]. The protein content of the suspension was determined according to Lowry et al. (1951) [43], with bovine serum albumin as a standard (Shimadzu UV1900 spectrophotometer, Shimadzu, Kyoto, Japan). The RBC ghosts were stored frozen (−70 °C) until analysis.

### 5.4. Determination of the RBC Na^+^/K^+^ ATPase Activity

For the assay procedure, a quantity equal to 300 µg protein was used. The relatively high quantity was reasoned by the fact that the abundance of the enzyme is relatively low in the red cells [44]. RBC ghosts were pre-incubated (10 min at 37 °C) in an incubation medium (92 mM TRIS-HCl (pH 7.4), 100 mM NaCl, 20 mM KCl, 5 mM MgCl_2_ and 1 mM EDTA) [45]. The reaction was started by the addition of 6 mM vanadate free ATP (disodium salt, Merck-Sigma Aldrich A26209). Incubations (37 °C at 30 min) were performed in the presence (2 mM) and absence of ouabain, a specific Na^+^/K^+^ ATPase inhibitor (ouabain octahydrate, Merck-Sigma Aldrich O3125). The reaction was stopped by addition of ice-cold trichloroacetic acid at a final concentration of 5%, and samples were centrifuged at 5000 g for 10 min at 4 °C. The phosphate liberation was determined from the supernatant and was given as the difference of the Pi liberation without and with ouabain, in nmol Pi/mg protein/h. The liberated Pi was measured photometrically (Shimadzu UV1900 spectrophotometer, Shimadzu, Kyoto, Japan), according to Hurst (1964) [46], in 10 mm path length optical glass cuvettes (Hellma Optik GmbH, Jena, Germany). All assays were performed in triplicate, and blanks were included in each run to determine the endogenous phosphate concentration and the non-enzymatic ATP breakdown (i.e., Pi liberation). The amount of phosphate was read from the standard curve prepared using known concentrations of KH_2_PO_4_, according to Beltowski and Wojcicka (2002) [47].

To ascertain that the ATP concentration in the medium is reaching the level of enzyme saturation, a simple test was performed, namely, the doubling of the enzyme quantity doubled the apparent enzyme activity, while the doubling of the ATP concentration did not alter it.

### 5.5. Determination of the RBC Membrane Fatty Acid Composition

The RBC ghost moiety (i.e., porous, lysed cells) not used for enzyme assay was directly lipid-extracted according to the method of Folch et al. (1957) [48] and the gained complex lipids’ (FA) composition was determined with gas chromatography (Shimadzu Nexis 2030, Kyoto, Japan), in the form of fatty acid methyl esters [36], after a separation on a Phenomenex Zebron ZB-Wax capillary column (30 m × 0.25 mm × 0.25 micrometer film, Phenomenex Inc., Torrance, CA, USA). The chromatographic evaluation was performed with the LabSolutions 5.93 software, using the PostRun module (Shimadzu, Kyoto, Japan) with manual peak integration. Fatty acid composition was expressed as weight % of total FA methyl esters. The identification of the FAs was performed based on the retention time of a CRM external standard (Supelco 37 Component FAME Mix, Merck-Sigma Aldrich, CRM47885).

### 5.6. Red Cell Antioxidant Status and Lipid Peroxidation

For the determination of lipid peroxidation and antioxidant status, the 3 × washed red blood cell samples (not lysed cells) were stored at −70 °C until analysis. Lipid peroxidation was determined by the quantification of malondialdehyde (MDA) levels with 2-thiobarbituric acid method in cell hemolysate [49] and the determination of conjugated dienes (CD) and trienes (CT) according to the photometric method of AOAC (1984) [50]. The concentration of reduced glutathione (GSH) was measured by the method of Sedlak and Lindsay (1968) [51] and the activity of glutathione peroxidase (GSPHx) according to Lawrence and Burk (1978) [52].

### 5.7. Statistical Analysis

For the comparison of the 3 group means (enzyme activity, initial and final bodyweight, fatty acid profile data within single rows) univariate (FBs concentration as grouping variable) analysis of variance (ANOVA) was used, with the LSD “post hoc” test for detailed inter-group differences. Pearson correlation was calculated between sodium pump activity and further biochemical variables, using individual data-pairs always. The extent of standard deviation was compared between groups with Levene’s F test. For all tests, significance level was set to *p* ≤ 0.05. IBM SPSS 20 for Windows (2010) [53] was used for the evaluation.

### 5.8. Ethical Issues

The experiments were carried out according to the regulations of the Hungarian Animal Protection Act. The allowance number for the studies was SOI/31/00308-10/2017 (date of approval: 27 March 2017).

## Figures and Tables

**Table 1:** Somatic traits of the experimental and control piglet groups (*n* = 6/group; data are group means ± SD (standard deviation) of 6 individual data; different uppercase letters indicate significant difference of means at *p* ≤ 0.05. Between group differences were compared with one-way ANOVA and LSD “post hoc” test; BW: bodyweight).

Group	Control				15 mg/kg				30 mg/kg			
Somatic Traits	Mean	±	SD		Mean	±	SD		Mean	±	SD	
BW initial (g)	12,980	±	1720		13,800	±	1200		13,800	±	1340	
BW final (g)	21,467	±	1735	a	23,067	±	1454	ab	23,367	±	629	b
cumulative feed intake (g)	19,759	±	2102		20,450	±	1352		20,382	±	1352	
lung (g)	227.2	±	45.0		237.1	±	43.0		253.0	±	70.0	
liver (g)	527.4	±	42.1		587.2	±	56.9		563.0	±	96.2	
kidney (g)	84.1	±	2.53		86.2	±	6.83		79.4	±	10.6	
pancreas (g)	50.0	±	6.39		57.1	±	7.62		56.5	±	9.81	
lung (% of BW)	1.06	±	0.20		1.03	±	0.15		1.09	±	0.30	
liver (% of BW)	2.48	±	0.38		2.55	±	0.23		2.42	±	0.45	
kindey (% of BW)	0.39	±	0.03	b	0.37	±	0.04	ab	0.34	±	0.05	a
pancreas (% of BW)	0.23	±	0.04		0.25	±	0.04		0.24	±	0.04	

**Table 2:** The red cell membrane fatty acid profile in the three piglet groups (*n* = 6/group; data are group means ± SD of 6 individual data; different uppercase indicate significant difference of means at *p* ≤ 0.05. Between group differences were compared with one-way ANOVA and LSD “post hoc” test; BW: bodyweight; MUFA: monounsaturated fatty acid; PUFA: polyunsaturated fatty acid).

Group	Control				15 mg/kg				30 mg/kg			
Red Cell Membrane FA Profile	Mean	±	SD		Mean	±	SD		Mean	±	SD	
C12:0	0.04	±	0.01	b	0.02	±	0.01	a	0.02	±	0.00	ab
C14:0	0.52	±	0.04		0.50	±	0.03		0.52	±	0.05	
C15:0	0.10	±	0.05		0.14	±	0.08		0.09	±	0.02	
C16:0	43.6	±	0.97	ab	42.6	±	1.69	a	44.5	±	1.33	b
C16:1n7	0.08	±	0.02		0.11	±	0.04		0.09	±	0.04	
C17:0	0.40	±	0.12		0.59	±	0.25		0.38	±	0.05	
C18:0	48.7	±	1.52		46.7	±	2.86		48.8	±	1.63	
C18:1n9c	3.90	±	1.64	ab	6.15	±	2.68	b	2.99	±	0.72	a
C18:1n7	0.23	±	0.10		0.33	±	0.18		0.17	±	0.03	
C18:2n6	0.96	±	0.70		1.54	±	1.29		0.37	±	0.11	
C18:3n3	0.02	±	0.00		0.02	±	0.01		0.03	±	0.00	
C20:0	0.45	±	0.03		0.43	±	0.05		0.43	±	0.03	
C20:1n9	0.02	±	0.00		0.02	±	0.01		0.02	±	0.00	
C20:2n6	0.02	±	0.01		0.02	±	0.01		0.01	±	0.01	
C20:3n6	0.02	±	0.01	ab	0.03	±	0.02	b	0.01	±	0.00	a
C21:0	0.03	±	0.02		0.03	±	0.02		0.04	±	0.02	
C20:4n6	0.25	±	0.17	ab	0.46	±	0.33	b	0.10	±	0.04	a
C22:0	0.07	±	0.01		0.06	±	0.01		0.06	±	0.00	
C22:6n3	0.15	±	0.05		0.18	±	0.04		0.15	±	0.06	
C24:1n9	0.08	±	0.08		0.09	±	0.08		0.08	±	0.07	
Σ saturated	93.1	±	0.91	ab	91.0	±	4.32	a	96.0	±	0.94	b
Σ unsaturated	6.92	±	0.91		8.95	±	4.32		3.96	±	0.94	
Σ MUFA	5.32	±	0.63	ab	6.70	±	2.92	b	3.34	±	0.78	a
Σ PUFA	1.40	±	0.87		2.25	±	1.68		0.63	±	0.17	
Σ n3	0.15	±	0.05		0.19	±	0.05		0.16	±	0.06	
Σ n6	1.25	±	0.88		2.06	±	1.64		0.49	±	0.16	
Σ n6/Σ n3	9.38	±	7.30		9.70	±	6.59		3.77	±	1.49	
Σ odd chain FA	0.50	±	0.17		0.72	±	0.33		0.47	±	0.06	

**Table 3:** Pearson correlation parameters between sodium pump activity and the fatty acid profile data. (Calculations were performed on pooled (i.e., 3 groups handled together as one, *n* = 18), individual data pairs were used for the analysis. Significance was set to *p* ≤ 0.05).

Compound	Sig.	Pearson Corr. Coeff.
C18:2 n6	0.017	−0.671
C20:2 n6	0.001	−0.821
C20:3 n6	0.022	−0.65
C20:4 n6	0.023	−0.648
Σ PUFA	0.012	−0.697
Σ n6	0.014	−0.683

**Table 4:** Diet proximate composition of the piglets.

Crude protein (%)	17.50
Crude fat (%)	3.30
Crude fiber (%)	3.70
Crude ash (%)	5.00
Lysine (g/kg)	1.11
Methionine (g/kg)	0.37
Ca (g/kg)	0.65
P (g/kg)	0.50
Na (g/kg)	0.18
DE (MJ/kg)	14.70
ME (MJ/kg)	14.10

## References

[1] Rheeder J.P., Marasas W.F.O., Vismer H.F. (2002). Production of fumonisin analogs by Fusarium species. Appl. Environ. Microbiol..

[2] Liu X., Fan L., Yin S., Chen H., Hu H. (2019). Molecular mechanisms of fumonisin B1-induced toxicities and its applications in the mechanism-based interventions. Toxicon.

[3] BIOMIN Holding GmbH, Getzersdorf, Austria. https://www.biomin.net/science-hub/world-mycotoxin-survey-impact-2020/.

[4] Haschek W.M., Gumprecht L.A., Smith G., Tumbleson M.E., Constable P.D. (2001). Fumonisin toxicosis in swine: An overview of porcine pulmonary edema and current perspectives. Environ. Health Perspect..

[5] Gumprecht L.A., Beasley V.R., Weigel R.M., Parker H.M., Tumbleson M.E., Bacon C.W., Meredith F.I., Haschek W.M. (1998). Development of fumonisin-induced hepatotoxicity and pulmonary edema in orally dosed swine: Morphological and biochemical alterations. Toxicol. Pathol..

[6] Stockmann-Juvala H., Savolainen K. (2008). A review of the toxic effects and mechanisms of action of fumonisin B 1. Hum. Exp. Toxicol..

[7] Loiseau N., Polizzi A., Dupuy A., Therville N., Rakotonirainy M., Loy J., Viadere J.L., Cossalter A.M., Bailly J.D., Puel O. (2015). New insights into the organ-specific adverse effects of fumonisin B1: Comparison between lung and liver. Arch. Toxicol..

[8] Wang E., Norred W.P., Bacon C.W., Riley R.T., Merrill A.H. (1991). Inhibition of sphingolipid biosynthesis by fumonisins. Implications for diseases associated with Fusarium moniliforme. J. Biol. Chem..

[9] Szabó A., Szabó-Fodor J., Fébel H., Romvári R., Kovács M. (2014). Individual and combined haematotoxic effects of fumonisin B1 and T-2 mycotoxins in rabbits. Food Chem. Toxicol..

[10] Haschek W.M., Motelin G., Ness D.K., Harlin K.S., Hall W.F., Vesonder R.F., Peterson R.E., Beasley V.R. (1992). Characterization of fumonisin toxicity in orally and intravenously dosed swine. Mycopathologia.

[11] Mays R.W., Siemers K.A., Fritz B.A., Lowe A.W., Van Meer G., Nelson W.J. (1995). Hierarchy of mechanisms involved in generating Na/K-ATPase polarity in MDCK epithelial cells. J. Cell Biol..

[12] Terciolo C., Bracarense A.P., Souto P.C.M.C., Cossalter A.M., Dopavogui L., Loiseau N., Oliveira C.A.F., Pinton P., Oswald I.P. (2019). Fumonisins at doses below EU regulatory limits induce histological alterations in piglets. Toxins.

[13] Ali O., Szabó-Fodor J., Fébel H., Mézes M., Balogh K., Glávits R., Kovács M., Zantomasi A., Szabó A. (2019). Porcine hepatic response to fumonisin b1 in a short exposure period: Fatty acid profile and clinical investigations. Toxins.

[14] Szabó A., Szabó-Fodor J., Kachlek M., Mézes M., Balogh K., Glávits R., Ali O., Zeebone Y.Y., Kovács M. (2018). Dose and exposure time-dependent renal and hepatic effects of intraperitoneally administered fumonisin B1 in rats. Toxins.

[15] Szabó A., Fébel H., Ali O., Kovács M. (2019). Fumonisin B1 induced compositional modifications of the renal and hepatic membrane lipids in rats–Dose and exposure time dependence. Food Addit. Contam. Part A Chem. Anal. Control. Expo. Risk Assess..

[16] Szabó A., Szabó-Fodor J., Fébel H., Mézes M., Repa I., Kovács M. (2016). Acute hepatic effects of low-dose fumonisin B1 in rats. Acta Vet. Hung..

[17] Yang C., Song G., Lim W. (2020). Effects of mycotoxin-contaminated feed on farm animals. J. Hazard. Mater..

[18] Chen Z., Chen H., Li X., Yuan Q., Su J., Yang L., Ning L., Lei H. (2019). Fumonisin B1 damages the barrier functions of porcine intestinal epithelial cells in vitro. J. Biochem. Mol. Toxicol..

[19] Riley R.T., Torres O., Matute J., Gregory S.G., Ashley-Koch A.E., Showker J.L., Mitchell T., Voss K.A., Maddox J.R., Gelineau-van Waes J.B. (2015). Evidence for fumonisin inhibition of ceramide synthase in humans consuming maize-based foods and living in high exposure communities in Guatemala. Mol. Nutr. Food Res..

[20] Riley R.T., Voss K.A., Yoo H.S., Gelderblom W.C.A., Merrill Jnr A.H. (1994). Mechanism of fumonisin toxicity and carcinogenesis. J. Food Prot..

[21] Zitomer N.C., Mitchell T., Voss K.A., Bondy G.S., Pruett S.T., Garnier-Amblard E.C., Liebeskind L.S., Park H., Wang E., Sulllards M.C. (2009). Ceramide synthase inhibition by fumonisin B1 causes accumulation of 1-deoxysphinganine. A novel category of bioactive 1-deoxysphingoid bases and 1-deoxydihydroceramides biosynthesized by mammalian cell lines and animals. J. Biol. Chem..

[22] Cabral L.M.P., Wengert M., Almeida F.G., Caruso-Neves C., Vieyra A., Einicker-Lamas M. (2010). Ceramide-activated protein kinases A and C zeta inhibit kidney proximal tubule cell Na+-ATPase. Arch. Biochem. Biophys..

[23] Kreydiyyeh S.I., Dakroub Z. (2014). Ceramide and its metabolites modulate time-dependently the activity of the Na+/K+ ATPase in HepG2 cells. Int. J. Biochem. Cell Biol..

[24] Hughes F.M., Bortner C.D., Purdy G.D., Cidlowski J.A. (1997). Intracellular K+ suppresses the activation of apoptosis in lymphocytes. J. Biol. Chem..

[25] Wu B.J., Hulbert A.J., Storlien L.H., Else P.L. (2004). Membrane lipids and sodium pumps of cattle and crocodiles: An experimental test of the membrane pacemaker theory of metabolism. Am. J. Physiol. Regul. Integr. Comp. Physiol..

[26] Zhang L., Zhang Z., Guo H., Wang Y. (2008). Na+/K+-ATPase-mediated signal transduction and Na+/K+-ATPase regulation. Fundam. Clin. Pharmacol..

[27] Colina C., Cervino V., Benaim G. (2002). Ceramide and sphingosine have an antagonistic effect on the plasma-membrane Ca2+-ATPase from human erythrocytes. Biochem. J..

[28] Carafoli E. (1994). Biogenesis: Plasma membrane calcium ATPase: 15 years of work on the purified enzyme 1. FASEB J..

[29] Nobel C.S.I., Aronson J.K., Van Den Dobbelsteen D.J., Slater A.F.G. (2000). Inhibition of Na+/K+-ATPase may be one mechanism contributing to potassium efflux and cell shrinkage in CD95-induced apoptosis. Apoptosis.

[30] Arrebola F., Zabiti S., Cañizares F.J., Cubero M.A., Crespo P.V., Fernández-Segura E. (2005). Changes in intracellular sodium, chlorine, and potassium concentrations in staurosporine-induced apoptosis. J. Cell. Physiol..

[31] Martínez-Mas J.V., Peinado-Onsurbe J., Ruiz-Montasell B., Felipe A., Casado F.J., Pastor-Anglada M. (1995). Na+,K+-ATPase expression during the early phase of liver growth after partial hepatectomy. FEBS Lett..

[32] Therien A.G., Blostein R. (2000). Mechanisms of sodium pump regulation. Am. J. Physiol.-Cell Physiol..

[33] Else P.L., Wu B.J., Storlien L.H., Hulbert A.J. (2003). Molecular activity of Na+,K+-ATPase relates to the packing of membrane lipids. Annals of the New York Academy of Sciences.

[34] Dwight J.F.S.J., Mendes Ribeiro A.C., Hendry B.M. (1992). Membrane incorporation of non-esterified fatty acids and effects on the sodium pump of human erythrocytes. Clin. Sci..

[35] Dwight J.F.S.J., Hendry B.M. (1995). Effects of membrane incorporation of short-chain phospholipids on sodium pump function in human erythrocytes. Clin. Chim. Acta.

[36] Christie W.W. (1982). A simple procedure for rapid transmethylation of glycerolipids and cholesteryl esters. J. Lipid Res..

[37] Yin J.J., Smith M.J., Eppley R.M., Page S.W., Sphon J.A. (1998). Effects of fumonisin B1 on lipid peroxidation in membranes. Biochim. Biophys. Acta-Biomembr..

[38] Fodor J., Balogh K., Weber M., Miklós M., Kametler L., Pósa R., Mamet R., Bauer J., Horn P., Kovács F. (2008). Absorption, distribution and elimination of fumonisin B(1) metabolites in weaned piglets. Food Addit. Contam. Part A Chem. Anal. Control. Expo. Risk Assess..

[39] Clemens M.R., Waller H.D. (1987). Lipid peroxidation in erythrocytes. Chem. Phys. Lipids.

[40] Fodor J., Kametier L., Kovács M. (2006). Practical aspects of fumonisin production under laboratory conditions. Mycotoxin Res..

[41] Bartók T., Tölgyesi L., Szekeres A., Varga M., Bartha R., Szécsi Á., Bartók M., Mesterházy Á. (2010). Detection and characterization of twenty-eight isomers of fumonisin B1 (FB1) mycotoxin in a solid rice culture infected with Fusarium verticillioides by reversed-phase high-performance liquid chromatography/electrospray ionization time-of-flight and ion trap mass spectrometry. Rapid Commun. Mass Spectrom..

[42] Shanmugasundaram K.R., Padmavathi C., Acharya S., Vidhyalakshmi N., Vijayan V.K. (1992). Exercise-induced cholesterol depletion and Na+,K+-ATPase activities in human red cell membrane. Exp. Physiol..

[43] Lowry O.H., Rosebrough N.J., Farr A.L., Randall R.J. (1951). Protein measurement with the Folin phenol reagent. J. Biol. Chem..

[44] Djemli-Shipkolye A., Raccah D., Pieroni G., Vague P., Coste T.C., Gerbi A. (2003). Differential effect of ω3 PUFA supplementations on Na,K-ATPase and Mg-ATPase activities: Possible role of the membrane ω6/ω3 ratio. J. Membr. Biol..

[45] Bedin M., Helena C., Estrella G., Ponzi D., Duarte D.V., Dutra-Filho C.S., Wyse A.T.S., Wajner M., Wannmacher C.M.D. (2001). Reduced Na+,K+-ATPase activity in erythrocyte membranes from patients with phenylketonuria. Pediatr. Res..

[46] Hurst R.O. (1964). The determination of nucleotide phosphorus with stannous chloride-hydrazine sulphate reagent. Can. J. Biochem. Physiol..

[47] Bełtowski J., Wójcicka G. (2002). Spectrophotometric method for the determination of renal ouabain-sensitive H+,K+-ATPase activity. Acta Biochim. Pol..

[48] Folch J., Lees M., Sloane Stanley G.H. (1957). A simple method for the isolation and purification of total lipides from animal tissues. J. Biol. Chem..

[49] Botsoglou N.A., Fletouris D.J., Papageorgiou G.E., Vassilopoulos V.N., Mantis A.J., Trakatellis A.G. (1994). Rapid, sensitive and specific thiobarbituric acid method for measuring lipid peroxidation in animal tissue, food and feedstuff samples. J. Agric. Food Chem..

[50] AOAC (1984). Official Methods of Analysis (28.054).

[51] Sedlak J., Lindsay R.H. (1968). Estimation of total, protein-bound, and nonprotein sulfhydryl groups in tissue with Ellman’s reagent. Anal. Biochem..

[52] Lawrence R.A., Burk R.F. (1978). Species, Tissue and Subcellular Distribution of Non Se-Dependent Glutathione Peroxidase Activity. J. Nutr..

[53] SPSS (2012). SPSS for Windows ver. 20.

